# Iranian Hydatid Disease Registry: Establishment and Implementation of a Neglected Tropical Disease Registry

**DOI:** 10.34172/aim.2023.54

**Published:** 2023-07-01

**Authors:** Saeid Nasibi, Shahnaz Mojarrab, Mohammad Reza Lashkarizadeh, Mohammad Shafiei, Ebrahim Saedi Dezaki, Hossein Mahmoudvand, Ardeshir Alizadeh, Alireza Mohammadzadeh, Seyed Jafar Adnani Sadati, Seyed Reza Mirbadie, Masoud Keighobadi, Shirzad Gholami, Saber Raeghi, Masoumeh Abbasi, Fatemeh Mohtasham, Mehrnaz Sadat Ravari, Mansour Dabirzadeh, Seyed Alireza Mosavi Anari, Hamed Mirjalali, Mohsen Aliakbarian, Mitra Abbasifard, Majid Fasihi Harandi

**Affiliations:** ^1^Research Center for Hydatid Disease in Iran, Kerman University of Medical Sciences, Kerman, Iran; ^2^Deputy of Research, Ministry of Health and Medical Education, Tehran. Iran; ^3^Department of Parasitology, School of Medicine, Shahrekord University of Medical Sciences, Shahrekord, Iran; ^4^Department of Laboratory Sciences, School of Allied Medicine, Lorestan University of Medical Sciences, Khorramabad, Iran; ^5^Disease and Health Outcomes Registry Department, Qazvin University of Medical Sciences, Qazvin, Iran; ^6^Department of Medical Microbiology and Immunology, Faculty of Medicine, Qom University of Medical Sciences, Qom, Iran; ^7^School of Medicine, Shahroud University of Medical Sciences, Shahroud, Iran; ^8^Toxoplasmosis Research Center, Communicable Diseases Institute, Iranian National Registry Center for Toxoplasmosis (INRCT), Mazandaran University of Medical Sciences, Sari, Iran; ^9^Molecular and Cell Biology Research Center, Mazandaran University of Medical Sciences, Sari, Iran; ^10^Department of Laboratory Sciences, Maragheh University of Medical Sciences, Maragheh, Iran; ^11^Department of Health Information Technology, Kermanshah University of Medical Sciences, Kermanshah, Kermanshah, Iran; ^12^Department of Parasitology and Mycology, School of Medicine, Zabol University of Medical Sciences, Zabol, Iran; ^13^Infectious and Tropical Diseases Research Center, Shahid Sadoughi University of Medical Sciences, Yazd, Iran; ^14^Foodborne and Waterborne Diseases Research Center, Research institute for Gastroenterology and Liver Diseases, Shahid Beheshti University of Medical Sciences, Tehran, Iran; ^15^Surgical Oncology Research Center, Imam Reza Hospital, Faculty of Medicine, Mashhad University of Medical Sciences, Mashhad, Iran; ^16^Department of Internal Medicine, Ali-Ibn-Abi-Talib Hospital, Rafsanjan University of Medical Sciences, Rafsanjan, Iran

**Keywords:** Biobanking, Disease surveillance, Echinococcosis, Hydatid cyst, National registry

## Abstract

**Background:**

Cystic echinococcosis (CE) or hydatid disease is a global public health concern which imposes considerable economic costs on the communities in endemic regions. CE surveillance data are not adequately reliable. The present study reports the development and outcomes of a CE registry in Iran.

**Methods:**

Hydatid Registry (HydatidReg) was initially established as a single-center registry in 2014 after the ethical approval of KMU. Following a call from MoHME to promote registry of different diseases and health outcomes, a call for participation was announced and all the Iranian Universities of Medical Sciences were requested to contribute to the registry. Subsequently, a nation-wide registry of hydatid disease was established in 2016. With a global perspective, HydatidReg joined the European Register of Cystic Echinococcosis (ERCE). A data collection form based on minimum dataset was designed and standard operating procedures (SOPs) were prepared to ensure standardized patient enrolment in the registry. A biobank system with two-dimensional barcoding was established along with HydatidReg for management and organization of biological specimens.

**Results:**

As of March 2021, a total of 690 patients were enrolled in the registry. HydatidReg registered 362 (17.3%) out of the total 2097 patients enrolled in ERCE. Quality control (QC) of the data demonstrated 91.2% completeness and 80% timeliness. In the biobank, 322 biological specimens from 184 CE patients have been deposited including 70 blood, 96 sera and 156 parasite materials.

**Conclusion:**

High-quality data in the HydatidReg registry provided opportunities for health professionals to improve quality of care and organize meaningful research.

## Introduction

 Hydatid disease or cystic echinococcosis (CE) is a zoonotic infection of humans caused by the larval stages of the small tapeworm *Echinococcus granulosus*. The disease is a public health concern and imposes considerable economic costs on the communities in endemic regions. CE is widespread from the South America and the Mediterranean countries of Europe, North Africa and the Middle East to the Central Asia and China. Iran is one of the hotspots of CE transmission in the world and human cases have been regularly reported from across the country.^1,2^

 Surgical intervention is the main therapeutic approach for CE management; however, according to current treatment guidelines, benzimidazole therapy is also indicated for patients with inoperable conditions and those with multiple/multi-organ involvement or with no clinical symptoms.^3^ The information provided by ultrasound images is valuable for the diagnosis and treatment of CE. The therapeutic approach for each patient is very much dependent on the cyst stage determined by the WHO-IWGE classification of hydatid cysts in which CE1 and CE2 are active well-developed cysts and CE4 and CE5 are degenerating inactive cysts.^4^

 Our knowledge of the prevalence and incidence of CE in different parts of the country is limited. Existing data indicate that the mean annual surgical incidence of CE in Iran is 1.6/10^5^ inhabitants ranging from 0.6/10^5^ to 2.6/10^5^ in different geographical areas of the country.^5^ Asymptomatic CE has been investigated using community-based ultrasound as well as serological surveys. Studies revealed an ultrasound prevalence of 0.2‒1.8%, while 7.3‒13.8% of the individuals were seropositive by ELISA.^1,6^ However routine CE surveillance data are not adequately reliable and timely.^7^

 Like most other zoonotic diseases, the routine CE surveillance system in Iran suffers from under-reporting and under-ascertainment.^8^ Alternatively, disease registry systems are more reliable tools for surveillance and management. Disease registry is a systematic platform for collection, storage, retrieval, analysis, and dissemination of information related to the individuals with a specific disease or condition.^9^ Registries offer more reliable and valid information, and provide rigorous evidence on the incidence, prevalence, natural history of the disease and the effectiveness of different treatment modalities. Large-scale multi-center research projects can be established on the data provided by disease registries. In addition, registries can contribute to generating hypotheses and/or evidence testable by randomized controlled trials (RCTs). Therefore, disease registry is a key element for understanding disease status, improving quality of care and implementing effective reliable disease surveillance and control interventions.^10^

 Health-related registries in Iran are in their infancy. Cancer registry was established and developed as the first disease registry in the country.^11^ Until recently, only a handful of disease registries were running at the regional or national level.^12^

 In 2014, to promote registry systems for different diseases and health outcomes, a call for proposals was launched by the Iranian Ministry of Health and Medical Education (MOHME). Subsequently, more than 70 proposals, mostly from medical universities and government institutions were approved and received financial support to initiate registries at the local or national level.^13^ This led to the establishment of 39 well-designed active registry systems in the country. The present study reports the development and outcomes of the CE registry in Iran.

## Materials and Methods

 MOHME has been promoting disease registries since 2014. The Ministry developed a special section in its administration entitled “National Program for Disease Registries” in the MOHME Deputy of Research and Technology. The purpose of the national program was to develop high-standard registries in the country. Regarding the high endemicity of CE in Iran, the Iranian Hydatid Disease Registry (HydatidReg) was initially established in 2014, following an official proposal to the MOHME by the Research Center for Hydatid Disease in Iran (RCHD), Kerman University of Medical Sciences. HydatidReg was primarily developed as a pilot registry program from 2011 to 2014 in Afzalipour Medical Center, the largest medical center in southeastern Iran, as a single-center, hospital-based clinical registry, and upgraded to the national scale in 2016.

 The HydatidReg proposal was reviewed and approved by the Ethics Review Committee of Kerman University of Medical Sciences, approval code IR.KMU.REC.1394.421. An informed consent form was filled by the patient before data collection. For the minors and disabled individuals, the forms were completed by his/her guardian and/or registry staff, respectively.

 Reviews were carried out on the relevant literature and disease registries for identification of the kind of information needed for the registry. Subsequently, a data collection form was designed including four sections: (*a*) general data of each patient (name, date of admission, discharge and operation) as well as demographic and epidemiological data (age, sex, ID code, occupation and location of residence); (*b*) past history of patient treatment and current therapeutic measures; (*c*) types of samples collected for biobanking; and (*d*) hydatid cyst characteristics including the number, size, location and the WHO-IWGE ultrasound classification of the cyst(s). Subsequently, the data collection form was moved to an online platform to facilitate data entry and to improve completeness and validity. Based on experiences from the early years of the registry, a stable user-friendly web-based registry software was developed in 2020 (https://hydatidreg.com).

 To achieve a successful registry and ensure that necessary information is collected for the registry while remaining feasible for the participants, a minimum necessary dataset was developed. A draft dataset was prepared and subjected to expert review and subsequently, the dataset was modified and finalized according to the experts’ comments. The ERCE form was used for validating the HydatidReg data collection form. A panel of clinical experts in hydatid disease were consulted for validating different parts of the form. To collect uniform data and to improve the value of information and data analysis, a data dictionary was developed by HydatidReg. The data dictionary provided clear, explicit and operational definitions of outcomes.

 Standard operating procedures (SOPs) were prepared to ensure standardized patient enrolment in the registry and achieving data with the same structure and quality. The SOPs were published in the guideline for hospital reports.

 Upon the endorsement of the registry by MOHME, a call for participation was announced and all the Universities of Medical Sciences were requested to contribute to the registry. Consequently, a memorandum of understanding was signed between the registry officials and the collaborating University. For each collaborating center a registry workshop and CME program was organized to train healthcare professionals about the basic principles and procedures of the hydatid disease registry as well as a brief course on the diagnosis and management of CE. HydatidReg was actively presented in the biannual national congress of parasitology and parasitic diseases as well as the national conferences on disease registry and health outcomes.

 Having a global approach in CE management and control, HydatidReg tried to be linked to international centers working on CE data registries. This improved the quality of patient registry and was an opportunity for exchange of ideas and experiments. Subsequently, HydatidReg joined the European Register of Cystic Echinococcosis (ERCE) in 2015 as the national coordinator of hydatid registry in the Iran.^14^ Forty-four centers are affiliated to the ERCE, and HydatidReg is among the seven non-European centers in ERCE.

 Data collection in HydatidReg was based on collecting data through face-to-face interviews with the hospitalized patients. When this method was not applicable, patients’ data were collected from the medical records. Surgery department and operating rooms, radiology department, infection control supervisor and pathology department were involved in patient registration upon diagnosis of hydatid cyst in a medical center.The hospital registry staff, responsible for collecting data from data sources, were notified about the potential candidates of hydatid cyst surgery and/or admission of a patient with initial CE diagnosis. The staff included physicians, nurses, health service management experts, ward secretaries, secretary of the department and parasitologists. The minimum requirements for data collection and entry personnel include necessary skills for interpreting medical records, patient consent, and basic IT skills.

 Another form of data collection was the retrospective data enquiries using ICD codes in the Hospital Information System (HIS). All the codes in ICD-10 corresponding to CE were used: B67.0, B67.1, B67.2, B67.3 and B67.4.

 Quality control (QC) was considered a key element in the registry. All HydatidReg records were quality checked in terms of completeness, validity, comparability and timeliness.

 One major activity of the registry staff was external QC, making sure that all patients occurring in the population were identified and included in the registry.^15-17^ Using the hospital information system, all CE patients admitted in the main hospital were enrolled in the registry. For those patients who underwent surgery in other public/private hospitals, a memorandum of understanding was signed between the registry and the partner hospitals. In addition, patient data were shared among local registries in neighboring provinces to make sure that no patient was left behind.

 Internal QC was implemented through the registry software. Most of the information items for each patient were defined as an obligatory field in the software; therefore, the chance of missing values in patient information was kept minimal.

 To avoid misdiagnosis and miscoding, all the registry data were cross-checked with the corresponding data in the HIS and pathology departments. All the codes corresponding to CE in ICD-10 were used. As most hospitals participating in the registry are major referral hospitals, CE patients are usually referred to the hospitals and it is assumed that the registered patients are representative of the general population. To increase the external validity of the data, major private hospitals with known cases of hydatid surgery were also included in the registry.

 In line with comparability of registry data, HydatidReg published a guideline for hospital reports to ensure using standardized definitions. Also, an “International consensus on terminology to be used in the field of echinococcoses” was used in all forms and SOPs of HydatidReg.^18^

 Timely data collection and processing is essential in CE registry, as patients admitted to the hospital for hydatid surgery stayed for a few days and the time for real-time patient registry is short. Usually, the hospital registry staff reviewed new admissions in the surgery wards once or twice weekly to identify new candidates for hydatid surgery. Moreover, nurses working in the operating room notify hospital registry staff as soon as a surgical case arrives. However, different medical centers demonstrated different qualities in timeliness and this is related to the size of the center, the number of hydatid admissions per week, and quality of personnel training.

 A biobank was established within HydatidReg with the aim of management and organization of biological specimens from patients and the parasite. A section for registering the type of biological samples was included in the data collection form. At present, four different biological specimens are included in the biobank, i.e., cyst membranes and/or protoscoleces, parasite DNA, patient serum and whole blood. To ensure specimen quality and quantity, the samples were preserved in -70 °C storage.SOPs were prepared and the staff were especially trained for this purpose. A two-dimensional barcoding system (Micronic Inc.) was used to provide private and reliable access to the samples. Also, patient information including age, location of residence, contact details, clinical data and cyst characteristics were recorded in a specially designed software. To enhance collaboration and facilitate sample transactions, a sample transfer form was developed for exchanging biobank specimens and data.

## Results

**Figure 1:**
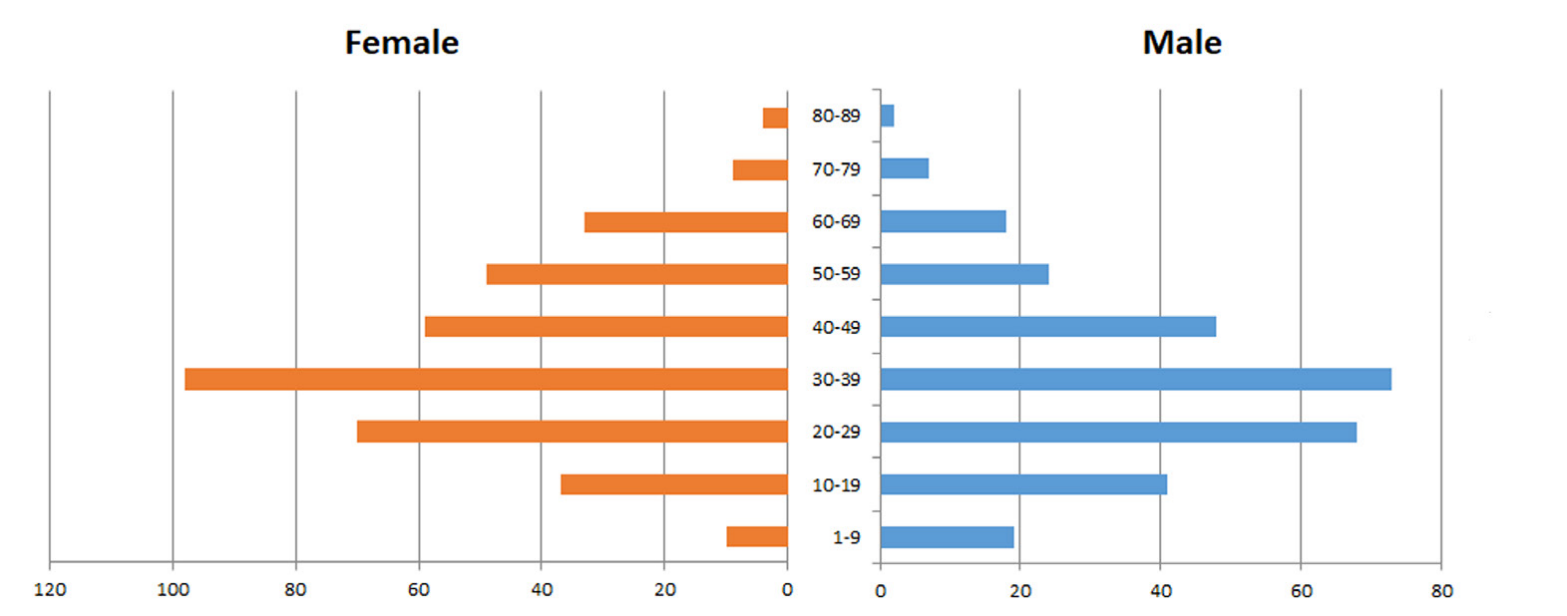
Age and Sex Distribution of 690 Patients Enrolled in the Iranian Hydatid Disease Registry, HydatidReg

 As of March 2021, a total of 690 patients were enrolled in the registry. Table 1 shows the provinces involved in the registry. More than 90% of the patients were registered from the Kerman, Lorestan, Shahrekord, Mazandaran and Qazvin provinces. Figure 1 demonstrates the age and sex distribution of patients enrolled in HydatidReg. The most affected age groups were 20‒49 years of age. No significant difference was found between the genders; although more female patients (55.2%) were registered than males (44.8%), in childhood ages, boys were more frequently affected than girls.

**Table 1:** Frequency Distribution of Patients Enrolled in HydatidReg According to the Universities Participating in the Registry

**Province**	**No. of Centers**	**Date of Affiliation**	**No. of Records**
Kerman	4	1395	421
Qazvin	1	1396	41
Lorestan	3	1396	67
Qom	7	1396	19
Chaharmahal and Bakhtiari	3	1396	63
Semnan (Shahroud)	2	1396	17
Mazandaran	1	1397	45
Other	8	-	17
Total	29	-	690

**Figure 2:**
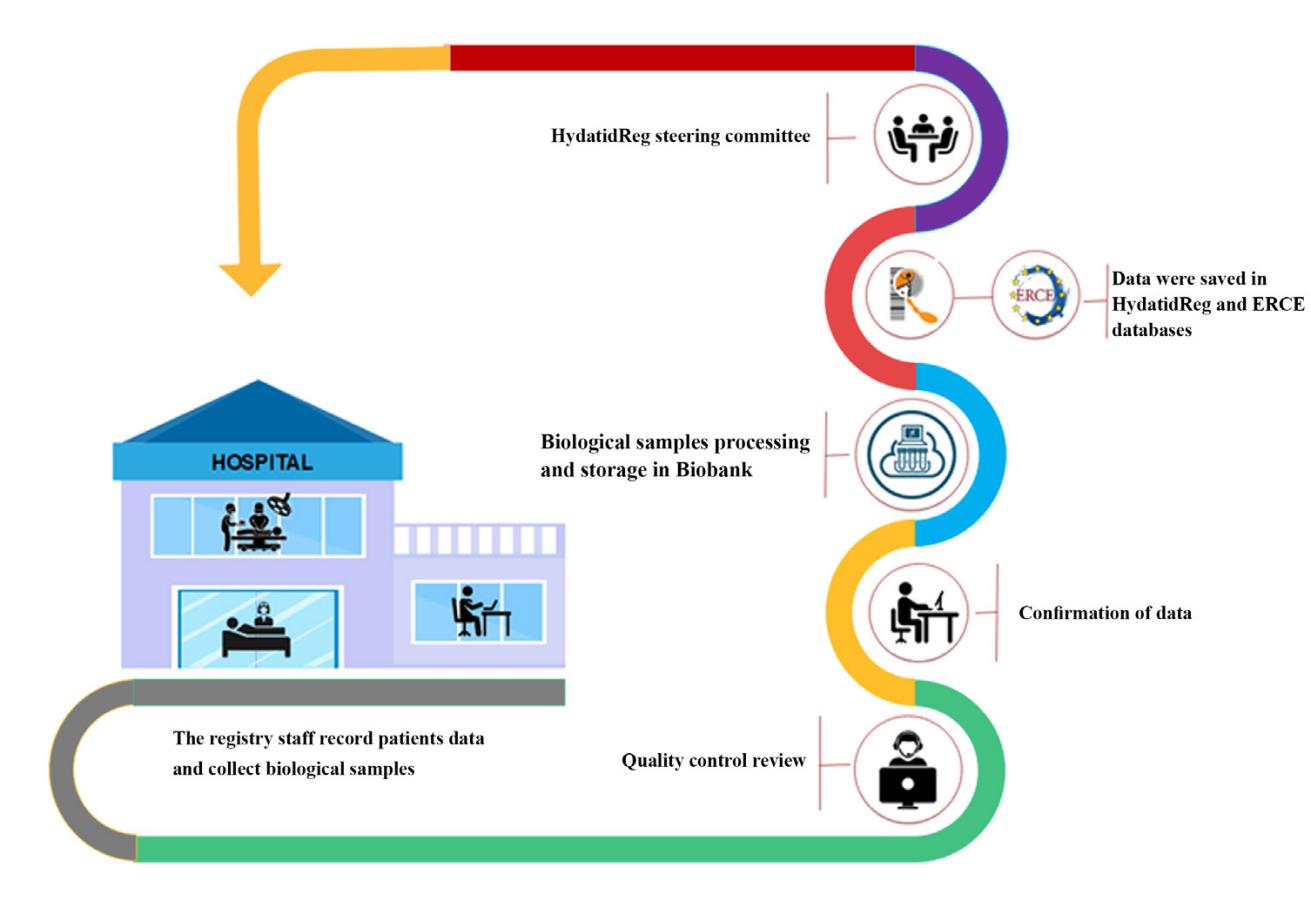
Workflow of the Iranian Hydatid Disease Registry, HydatidReg, from CE Patient Data Collection to Quality Control and Data Validation

Figure 2 demonstrates the workflow of the CE patient registry. According to the SOPs and the guideline, 690 patients were enrolled in the registry. In the 11^th^ National Congress of Parasitology and Parasitic Diseases held in Urmia in October 2019, special lectures were dedicated to promote the CE registry in the country. Moreover, the Iranian Society of Parasitology encouraged members to establish registry systems for endemic parasitic diseases in Iran.

 Eight CME programs were organized in 2019 in eight different centers including Isfahan, Kerman, Qom, Qazvin, Kermanshah, Khorramabad, Shahrekord and Bam. Surgeons, radiologists, medical parasitologists, internal medicine and infectious diseases specialists, nurses and communicable diseases surveillance officials generally participated in the CME programs. With the emergence of COVID-19, CME programs on hydatid registry have shifted to online meetings in 2020 with the participation of a broader audience.

 In the international context, as of March 2019, HydatidReg registered 362 (17.3%) out of the 2097 total number of patients enrolled in ERCE. In addition, HydatidReg facilitated the extension of ERCE to include one center from Afghanistan, thus 38 patients born in Afghanistan were registered in ERCE from Iran. A joint meeting was held in 2019 between Iranian clinical specialists and hepatobiliary surgeons working with the CE control program in the Rio Negro province, Argentina, in which a training course on Focused Assessment with Sonography for Echinococcosis (FASE) was presented and discussed.

 QC of the data demonstrated 91.2% completeness and 80% timeliness. Analysis of HydatidReg data indicated that 8.8% of the records have at least one missing value; therefore, the completeness was estimated at 91.2%. A relatively suitable timeliness was found in recording data. Eighty percent of the patients were registered in HydatidReg within three days of hydatid surgery.

 The hydatid biobank system was established in Afzalipour Medical Center in 2018. As of September 2020, 322 biological specimens from 184 CE patients have been deposited in the biobank including 70 blood samples, 96 sera and 156 parasite materials. Other centers affiliated to HydatidReg have not developed biobanks yet; however, based on the agreement for the transfer of biological materials, hydatid specimens could be exchanged between two or more centers.

## Discussion

 We present the processes in which the national echinococcosis registry has been established and developed in Iran. During the past years since 2014, the registry has developed from a single-center to a nationwide registry. Echinococcosis registry is relatively a recent development in the world. At the national level, the FrancEchino network was one of the first attempts for systematic surveillance of alveolar echinococcosis in France. Established in 2004, the FrancEchino network performed data collection within the framework of an agreement between the French Institute for Public Health Surveillance and the FrancEchino network. This allowed the FrancEchino registry to serve as a reliable source of epidemiological and clinical studies.^19,20^

 In the international context, the European Echinococcosis Registry (EurEchinoReg) as a surveillance network for human alveolar echinococcosis was the first registry of its kind established in 1998. EurEchinoReg created a network of 11 reporting centers across nine European countries of western and central Europe as well as Turkey, assessing human alveolar echinococcosis.^21^

 For CE, the Italian registry of CE was a multicenter system of CE patient registry, established in 2012 as a network of Italian health centers. The registry provided valuable information on the epidemiology of CE in Italy at the national level.^22^

 Subsequently in October 2014, the ERCE, as a prospective, observational, multicenter register of patients was launched as part of the HERACLES project.^14,23^

 In Iran, the CE surveillance system has been suffering from under-reporting and under-ascertainment. CE data from patients around the country were largely scattered and no systematic data collection system was available in the country. Information was mainly published from retrospective description of hospital records. The need for an effective and reliable CE information system lead to the establishment of HydatidReg in 2014. In 2015, HydatidReg joined the ERCE as the national coordinator of hydatid registry in Iran. Few countries in the endemic and hyperendemic regions of the world have ongoing active CE registries.^2^ Unfortunately, CE registry has not yet been established in any country in the region. HydatidReg is seeking collaborations in the MENA region to develop and improve the CE information and surveillance system.^24^

 Establishment of the biobank in the registry provided opportunities for the faculties and graduate students in basic and clinical departments to organize meaningful research projects on CE. After establishing the biobank within HydatidReg, improvements in the quality of research and training are expected as a result of access to high quality samples in a shorter time period.

 Disease registries are in their infancy in Iran. In particular, registry of neglected tropical diseases are facing major challenges and pitfalls in developing countries. These challenges can be categorized as executive, clinical and technical. The most prominent challenge in HydatidReg is the proactive participation of the university/public/private centers. Lack of an efficient patient referral system in the country, inefficient active CE surveillance system and reliability of the data retrieved from hospital records are among the major challenges of HydatidReg. As reflected in findings of 91.2% completeness of the records, no WHO ultrasound classification was provided for most registered patients. Widespread adoption of the WHO-IWGE ultrasound classification of CE is another goal of HydatidReg to improve the quality of care and suitable management of CE.

 Some other challenges within the registry need to be addressed in HydatidReg, including missing patients/data, contradictory post-operation diagnoses, and issues related to the HIS data retrievability as well as encouraging other centers within HydatidReg to develop biobanks and a well-organized CE sample/data management system. QC analysis indicates 20% time delay in patient registry, presenting the challenge of on-time registration. Finally, we need continuous public/professional education to promote CE registry and biobanking, particularly in the endemic areas.

## Conclusion

 As a young registry in its early steps, the national registry of echinococcosis in Iran (HydatidReg), has provided more reliable disease surveillance and management data for better understanding of CE status in the country and improving quality of patients care. Background information provided by the registry can pave the way for planning an effective CE control program, to be successfully implemented throughout the country. We hope WHO Eastern Mediterranean Regional Office can provide detailed guideline for cystic echinococcosis control for all the 22 CE-endemic countries in the region in accordance with the WHO road map for neglected tropical diseases 2021–2030.

## References

[1] Deplazes P, Rinaldi L, Alvarez Rojas CA, Torgerson PR, Fasihi Harandi M, Romig T (2017). Global distribution of alveolar and cystic echinococcosis. Adv Parasitol.

[2] Borhani M, Fathi S, Darabi E, Jalousian F, Simsek S, Ahmed H (2021). Echinococcoses in Iran, Turkey, and Pakistan: old diseases in the new millennium. Clin Microbiol Rev.

[3] Kern P, Menezes da Silva A, Akhan O, Müllhaupt B, Vizcaychipi KA, Budke C (2017). The echinococcoses: diagnosis, clinical management and burden of disease. Adv Parasitol.

[4] Wen H, Vuitton L, Tuxun T, Li J, Vuitton DA, Zhang W (2019). Echinococcosis: advances in the 21st century. Clin Microbiol Rev.

[5] Fasihi Harandi M, Budke CM, Rostami S (2012). The monetary burden of cystic echinococcosis in Iran. PLoS Negl Trop Dis.

[6] Fasihi Harandi M, Moazezi SS, Saba M, Grimm F, Kamyabi H, Sheikhzadeh F (2011). Sonographical and serological survey of human cystic echinococcosis and analysis of risk factors associated with seroconversion in rural communities of Kerman, Iran. Zoonoses Public Health.

[7] Ebrahimipour M, Budke CM, Fasihi Harandi M (2020). Control of cystic echinococcosis in Iran: where do we stand?. Trends Parasitol.

[8] Afsar Kazerooni P, Fararouei M, Nejat M, Akbarpoor M, Sedaghat Z (2018). Under-ascertainment, under-reporting and timeliness of Iranian communicable disease surveillance system for zoonotic diseases. Public Health.

[9] Gliklich RE, Dreyer NA, Leavy MB. Registries for Evaluating Patient Outcomes: A User’s Guide [Internet]. 3rd ed. Rockville, MD: Agency for Healthcare Research and Quality; 2014. Available from: https://www.ncbi.nlm.nih.gov/books/NBK208615/. 24945055

[10] Hoque DM, Kumari V, Ruseckaite R, Romero L, Evans SM (2016). Impact of clinical registries on quality of patient care and health outcomes: protocol for a systematic review. BMJ Open.

[11] Etemadi A, Sadjadi A, Semnani S, Nouraie SM, Khademi H, Bahadori M (2008). Cancer registry in Iran: a brief overview. Arch Iran Med.

[12] Aghamohammadi A, Moein M, Farhoudi A, Pourpak Z, Rezaei N, Abolmaali K (2002). Primary immunodeficiency in Iran: first report of the National Registry of PID in Children and Adults. J Clin Immunol.

[13] Mojarrab S, Rafei A, Akhondzadeh S, Jeddian A, Jafarpour M, Zendehdel K (2017). Diseases and health outcomes registry systems in IR Iran: successful initiative to improve public health programs, quality of care, and biomedical research. Arch Iran Med.

[14] Rossi P, Tamarozzi F, Galati F, Pozio E, Akhan O, Cretu CM (2016). The first meeting of the European Register of Cystic Echinococcosis (ERCE). Parasit Vectors.

[15] McLeod PJ, Meagher TW, Steinert Y, Boudreau D (2000). Using focus groups to design a valid questionnaire. Acad Med.

[16] Arts DG, De Keizer NF, Scheffer GJ (2002). Defining and improving data quality in medical registries: a literature review, case study, and generic framework. J Am Med Inform Assoc.

[17] Pearson ML, Ganz PA, McGuigan K, Malin JR, Adams J, Kahn KL (2002). The case identification challenge in measuring quality of cancer care. J Clin Oncol.

[18] Vuitton DA, McManus DP, Rogan MT, Romig T, Gottstein B, Naidich A (2020). International consensus on terminology to be used in the field of echinococcoses. Parasite.

[19] Charbonnier A, Knapp J, Demonmerot F, Bresson-Hadni S, Raoul F, Grenouillet F (2014). A new data management system for the French National Registry of human alveolar echinococcosis cases. Parasite.

[20] Chauchet A, Grenouillet F, Knapp J, Richou C, Delabrousse E, Dentan C (2014). Increased incidence and characteristics of alveolar echinococcosis in patients with immunosuppression-associated conditions. Clin Infect Dis.

[21] Kern P, Bardonnet K, Renner E, Auer H, Pawlowski Z, Ammann RW (2003). European echinococcosis registry: human alveolar echinococcosis, Europe, 1982-2000. Emerg Infect Dis.

[22] Tamarozzi F, Rossi P, Galati F, Mariconti M, Nicoletti GJ, Rinaldi F (2015). The Italian registry of cystic echinococcosis (RIEC): the first prospective registry with a European future. Euro Surveill.

[23] Rossi P, Tamarozzi F, Galati F, Akhan O, Cretu CM, Vutova K (2020). The European Register of Cystic Echinococcosis, ERCE: state-of-the-art five years after its launch. Parasit Vectors.

[24] Borhani M, Fathi S, Lahmar S, Ahmed H, Abdulhameed MF, Fasihi Harandi M (2020). Cystic echinococcosis in the Eastern Mediterranean region: neglected and prevailing!. PLoS Negl Trop Dis.

